# Editorial: Prevention of and early intervention for behavioral health disorders in people with epilepsy

**DOI:** 10.3389/fneur.2025.1762678

**Published:** 2026-01-13

**Authors:** Janelle L. Wagner, Braxton B. Wannamaker, Venus Tang, Julie DesMarteau, Jonathan J. Halford

**Affiliations:** 1 College of Nursing, Department of Pediatrics, Medical University of South Carolina, Charleston, SC, United States; 2 Department of Neurology, Medical University of South Carolina, Charleston, SC, United States; 3 Division of Neurosurgery, Department of Surgery, Faculty of Medicine, The Chinese University of Hong Kong, Sha Tin, Hong Kong SAR, China; 4 Department of Clinical Psychology, Prince of Wales Hospital, Hospital Authority, Sha Tin, Hong Kong SAR, China; 5 Department of Child Neurology, University of Rochester Medical Center, Rochester, NY, United States; 6 Neurology Service, Ralph H. Johnson Veterans Affairs Medical Center, Charleston, SC, United States

**Keywords:** behavioral health, behavioral health care, epilepsy, mental health, psychological distress

Persons with epilepsy (PWE) are at three–five times higher risk for behavioral health concerns, under which we summarize here as anxiety, depression, attention-deficit/hyperactivity disorder, substance use, cognitive disorders, etc., compared to those without epilepsy (1, 2). These concerns are more detrimental to health-related quality of life (HRQOL) and health outcomes than seizure-specific factors (3). Studies have examined the potential shared neuropathophysiology and genetics of epilepsy and behavioral health symptoms (4, 5). To further elucidate this complicated relationship, recent studies have suggested a bidirectional relationship between cognitive (e.g., memory, processing speed, IQ, etc.) and mental or behavioral health (e.g., anxiety, depression, substance use) symptoms as well a common mechanism for these co-occurring symptoms in PWE (6). These behavioral health concerns often remain undetected, with nearly two-thirds not receiving indicated behavioral health care [e.g., psychoeducation, cognitive-behavioral therapy (CBT), etc.], resulting in higher rates of unmet needs compared to other populations (7–10). Indeed, insufficiently managed behavioral health concerns in PWE are associated with higher rates of health care utilization and poorer health outcomes (11–13).

To address these gaps, the goal of this Research Topic was to promote the prevention of and early intervention for behavioral health concerns in PWE. Seven papers included in this Research Topic support aspects of neurology provider-led integrated behavioral health care (14), including promotion of identifying behavioral health issues through patient discussions and screening (Chan and Wong; Huang et al.), referral for behavioral health treatment (Kiriakopoulos et al.), managing anti-seizure medications (ASMs) and their side effects (Xu et al.), and providing psychoeducation to PWE (Hao et al.; Tang et al.) and the community (Younes et al.).

Screening for anxiety and depression in routine epilepsy encounters has been identified as a quality indicator by the American Academy of Neurology (AAN) (15) and highlighted in consensus recommendations (16); however, few screening instruments have been developed and validated for PWE (17). In this special report, Chan and Wong developed and validated the traditional-Chinese version of the well-established Epilepsy Anxiety Survey Instrument (EASI) and its brief screener (brEASI). They demonstrated strong reliability (i.e., internal consistency, test re-test) and construct validity (i.e., factor structure, convergent, divergent), providing support for use of these screening measures. Huang et al. found an increased odds of 26% for epilepsy in those adults who reported smoking tobacco, suggesting an association between nicotine use and seizures. Further investigation into the biological mechanisms underlying this association is warranted, particularly with relation to sex hormones, as well as potential psychosocial explanations (e.g., stress reduction, insomnia). This study and other recent investigations indicate the importance of screening for substance use in PWE, especially given the potential interference of substances with ASMs and neurological functioning.

When significant behavioral health concerns are identified, neurology providers are encouraged to consider: (1) treating mild anxiety and depression according to consensus recommendations, typically with SSRIs (16, 18), or (2) referring to a behavioral health care specialist (e.g., psychiatrist, psychologist, social worker, etc.) for moderate to severe symptoms. In this Research Topic, Kiriakopoulos et al. examined the benefit of HOme Based Self-management and COgnitive Training CHanges lives (HOBSCOTCH). Results were promising with teens and adults aged 16–83 years reporting significantly improved HRQOL and cognition following the 8-week program.

Providing psychoeducation on the etiologies and treatments for behavioral health concerns in PWE is critical to improve patient understanding. Two papers address the possible underlying pathophysiology of seizures and co-occurring behavioral health symptoms. Hao et al. studied stereo-electroencephalography (SEEG) in five patients with drug-resistant epilepsy who exhibited slapping automatisms, including one person with comorbid obsessive-compulsive disorder (OCD). Preliminary findings highlight a potential shared orbitofrontal-striatal-pallidal-thalamic network between frontal/temporal epilepsy, slapping automatism, and OCD. In addition, Tang et al. examined circadian rhythms using actigraphy. Compared to the general population, PWE showed significant differences in intraday stability (IS), or the fidelity of the pattern of rest to activity with respect to the light-dark cycle, and the peak activity period over 10 consecutive hours (M10), suggesting a disruption in the 24-h rest-activity circadian rhythm. While additional research is necessary, these findings may shed light on the seizure susceptibility and sleep difficulties that PWE experience. The importance of sleep-wake behaviors for PWE is a critical component of psychoeducation.

A broader framework for psychoeducation includes community-based education on the overlap between epilepsy and behavioral health symptoms to reduce stigma and promote awareness. Younes et al. assessed the knowledge, awareness, and attitudes toward epilepsy. Cultural adaptations included measures translated into Arabic and selection of measures that inquired about epilepsy in the context of Middle Eastern cultural beliefs. Results revealed that most respondents had a good knowledge of epilepsy (87%) and positive attitude toward patients with epilepsy (88%). However, 38.5% were “frightened” over individuals having seizures, suggesting the need for additional psychoeducation about how to respond to a seizure.

Finally, managing ASMs, the primary and first-line treatment for seizures, is a standard aspect of epilepsy care but can have undesired side effects. In a systematic review and meta-analysis, Xu et al. synthesize the data on use of ASMs in pregnant women with epilepsy. Six cohort studies showed that *in-utero* exposure to valproate was associated with an increased risk of autism spectrum disorder (ASD) and ADHD in their children.

These seven papers cover a wide range of topics related to the prevention and treatment of behavioral health concerns in PWE, contributing unique and important findings to epilepsy research. However, future studies are needed to further elucidate the shared pathophysiological and/or genetic underpinnings of epilepsy and behavioral health symptoms. Designs that employ randomized controlled trials (RCTs) to examine the benefit of self-management and other behavioral health interventions are indicated. Integrating behavioral health services into routine epilepsy care has been shown to improve HRQOL and reduce health care utilization without increasing health care costs (19, 20). At a minimum, integrated care should include, at diagnosis and over the course of epilepsy, psychoeducation about the co-occurrence of behavioral health concerns and what interventions have provided benefit. In addition, screening for common concerns, such as anxiety, depression, and ADHD during routine epilepsy visits will identify concerns early and promote participation in behavioral health intervention, which may improve seizure outcomes. Ideally, provision of these interventions occurs within epilepsy clinics to foster communication among interprofessional providers, promote comprehensive care, and reduce stigma.
